# Investigation of the High Mobility IGZO Thin Films by Using Co-Sputtering Method

**DOI:** 10.3390/ma8052769

**Published:** 2015-05-21

**Authors:** Chao-Ming Hsu, Wen-Cheng Tzou, Cheng-Fu Yang, Yu-Jhen Liou

**Affiliations:** 1 Department of Mechanical Engineering, National Kaohsiung University of Applied Science, No. 415 Chien Kung Road, Kaohsiung 807, Taiwan; E-Mail: jammy@kuas.edu.tw; 2 Department of Electro-Optical Engineering, Southern Taiwan University, No. 1, Nan-Tai Street, Yungkang Dist., Tainan City 710, Taiwan; E-Mail: wjtzou@mail.stust.edu.tw; 3 Department of Chemical and Materials Engineering, National University of Kaohsiung, No. 700 Kaohsiung University Road, Nan-Tzu District, Kaohsiung 811, Taiwan; E-Mail: sonic7838@hotmail.com

**Keywords:** IGZO, co-sputtering method, deposition power, SIMS

## Abstract

High transmittance ratio in visible range, low resistivity, and high mobility of IGZO thin films were prepared at room temperature for 30 min by co-sputtering of Zn_2_Ga_2_O_5_ (Ga_2_O_3_ + 2 ZnO, GZO) ceramic and In_2_O_3_ ceramic at the same time. The deposition power of pure In_2_O_3_ ceramic target was fixed at 100 W and the deposition power of GZO ceramic target was changed from 80 W to 140 W. We chose to investigate the deposition power of GZO ceramic target on the properties of IGZO thin films. From the SEM observations, all of the deposited IGZO thin films showed a very smooth and featureless surface. From the measurements of XRD patterns, only the amorphous structure was observed. We aimed to show that the deposition power of GZO ceramic target had large effect on the *E_g_* values, Hall mobility, carrier concentration, and resistivity of IGZO thin films. Secondary ion mass spectrometry (SIMS) analysis in the thicknesses’ profile of IGZO thin films found that In and Ga elements were uniform distribution and Zn element were non-uniform distribution. The SIMS analysis results also showed the concentrations of Ga and Zn elements increased and the concentrations of In element was almost unchanged with increasing deposition power.

## 1. Introduction

The typical plasma enhanced chemical vapor deposition (PECVD) hydrogenated amorphous silicon (α-Si:H) thin-film transistors (TFTs) are mainly applied for flat panel displays (FPDs), such as electronic papers (e-papers), organic light-emitting-diode displays (OLEDs), and liquid crystal displays (LCDs). Even α-Si:H TFTs have sub-threshold swing of 0.3~0.4 V/decade, off-state drain current (I_Doff_) below 10^−13^ A, and on-to-off ratio about 10^7^, they have the shortcoming of low field-effect mobility (μ_eff_) of about 0.6~0.8 cm^2^/V-s and poor transparency [1]. To address this issue, several n-type transparent amorphous oxide semiconductors (TAOSs), which exhibit high mobility, excellent uniformity, good transparency and applicability for the low-temperature process (for polymer or plastic substrate), and have potential to serve as active layer in TFTs [2,3]. Recently, conventional amorphous or polycrystalline transparent conduction oxide semiconductors (TCOs) have been proposed as alternative channel materials, because they exhibit excellent optical transparency and good TFTs performance in ambient conditions. However, the grain boundaries of TCOs could affect device properties, such as uniformity and stability, over large areas. For that, over the last several years, there has been great interest in TFTs made of TCOs-based TAOSs thin films. Recently, several n-type TAOSs thin films, such as ZnO [4], Al-Sn-Zn-O (ASZO) [5], and In-Ga-Zn-O (IGZO) [6], have received a considerable attention in the large-area FPD industry since they may overcome the difficulties encountered in the amorphous α-Si:H and polycrystalline silicon TFTs technologies [7].

This is mainly due to TAOSs’ thin-film transistors having unique advantages, such as transparency in visible light region, large-area uniform deposition at low temperature, and high carrier mobility. IGZO thin films are a semiconducting material and they can be used as the TFTs’ backplane of FPDs because IGZO-TFTs’ mobility is higher than that of amorphous silicon. The α-IGZO thin films could be deposited on polyethylene terephthalate at room temperature and exhibited high Hall effect mobility [8]. IGZO-TFTs were first developed by Professor H. Hosono’s group at Tokyo Institute of Technology and Japan Science and Technology Agency (JST) in 2003 for crystalline IGZO-TFTs [7] and in 2004 for amorphous IGZO (α-IGZO)-TFTs [8]. They found that an n-type amorphous In-Ga-Zn-O with a molar ratio 1:1:1 is preferred for fabricating electronic devices because it has a reasonably large Hall mobility (>15 cm^2^/V-s). Zan *et al.* reported that they utilized self-organized polystyrene spheres with a diameter of 200 nm to fabricate a porous gate structure and Ar plasma treatment through the porous gate performed dot-like doping on α-IGZO channel region. They fabricated a top-gate self-aligneda-IGZO TFT with an effective field-effect mobility as 79 cm^2^/V-s, they also reported that an intrinsic IGZO thin film had electron mobility as 39.6 cm^2^/V-s [9]. Bak et al used IGZO to fabricate the top-gate structured TFTs and the mobility of IGZO thin films was in the range of 11.8 cm^2^/V-s~14.8 cm^2^/V-s under different bias voltage [10]. Therefore, IGZO-TFTs can improve operation speed, resolution, and size of FDPs, and they are also considered as one of the most promising TFTs to drive OLED displays. As we know, various techniques were investigated for growth of IGZO thin films, such as electron beam evaporation, ion beam assisted deposition, and ion implantation. In particular, Jeong *et al.* obtained IGZO thin films by co-depositing the Ga:In_2_O_3_ and Zn:In_2_O_3_ targets to deposit the Ga and Zn co-doped In_2_O_3_ electrode at room temperature [11].

In the past, K. Nomura *et al.* presented that IGZO thin films are composed of alternating stacks of InO_2_^−^ and GaO(ZnO)^+^ layers [12]. They found that the In_2_O_3_ concentration in IGZO thin films has a large effect on the properties of IGZO thin films, especially in the electrical properties. In this study, the Zn_2_Ga_2_O_5_ and In_2_O_3_ ceramic targets were prepared separately and the two ceramic targets were used to deposit IGZO thin films by co-sputtering method at room temperature on glass substrates. We believed that as the deposition power of In_2_O_3_ was fixed, the concentration of the In_2_O_3_ in IGZO thin films could be controlled by changing the deposition power of GZO ceramic target. We systematically examined the crystallization, optical, and electrical properties and surface and cross-section morphologies of IGZO thin films as a function of deposition power of GZO ceramic target. Importantly, we showed that as the deposition power of GZO ceramic target was changed, the co-deposited IGZO thin films had the high mobility of 11.0 cm^2^/V-s~163.4 cm^2^/V-s.

## 2. Experimental Section

Ga_2_O_3_ powder (99.99%) was mixed with ZnO powder (99.99%) to form the Ga_2_O_3_-2ZnO composition (abbreviated as GZO). After being dried and ground, the GZO powder was calcined at 800 °C for 1 h, and ground again. GZO powder and In_2_O_3_ powder were mixed with polyvinylalcohol (PVA) as binder, and then the mixed powders were uniaxially pressed into pellets of 5 mm thickness and 54 mm diameter using a steel die. After being debindered, the GZO pellet and In_2_O_3_ pellet were sintered at 1200 °C and 1250 °C, respectively, for 2 h. Glass substrates (Corning 1737) with an area of 2 × 2 cm^2^ were cleaned ultrasonically with isopropyl alcohol (IPA) and deionized (DI) water, and dried under a blown nitrogen gas. Then GZO and pure In_2_O_3_ ceramic targets were used to co-deposit the IGZO thin films. Deposition power of In_2_O_3_ ceramic target was 100 W and deposition power of GZO ceramic target was changed from 80 W to 140 W, respectively, room temperature (RT) and 30 min were used as deposition temperature and deposition time. The base pressure of sputtering chamber was below 5 × 10^−6^ Torr and the working pressure was maintained at 3 × 10^−3^ Torr in pure Ar (99.99%) ambient. Thickness and surface morphology of IGZO thin films were measured using a field emission scanning electron microscopy (FESEM), and their crystalline structures were measured using X-ray diffraction (XRD) patterns with Cu Kα radiation (λ = 1.5418 Å). Energy dispersive spectrometer (EDS) and secondary ion mass spectrometry (SIMS) analyses were used to find the concentration variations of In, Ga, and Zn elements in the depth profile of IGZO thin films. Atomic Force Microscopy (AFM Analysis, Bruker, Germany) was used to measure surface topography and surface roughness of IGZO thin films. The optical transmission spectrum was recorded using a Hitachi U-3300 UV-V is spectrophotometer in the 250–1000 nm wavelength range. In the past, determination of the optical band gap (*E_g_*) values was often necessary to develop the electronic band structure of a thin-film material. A Tauc plot is one method of determining the *E_g_* values in semiconductors. However, as the Tauc plot is used, the *E_g_* values of thin films are extracted from the data of absorption coefficient as a function of photon energy (*hν)*. As the Tauc plot is used, the *E_g_* values of IGZO thin films can be determined using the relation in Equation (1):

(α*hv*)^2^ = *c*(*hν* − *E_g_*) (1)

where α is the optical absorption coefficient, *c* is the constant for direct transition, *h* is Planck’s constant, and *ν* is the frequency of the incident photon [13]. While the Hall-effect coefficient of IGZO thin films was measured using a Bio-Rad Hall set-up.

## 3. Results and Discussion

**Figure 1:**
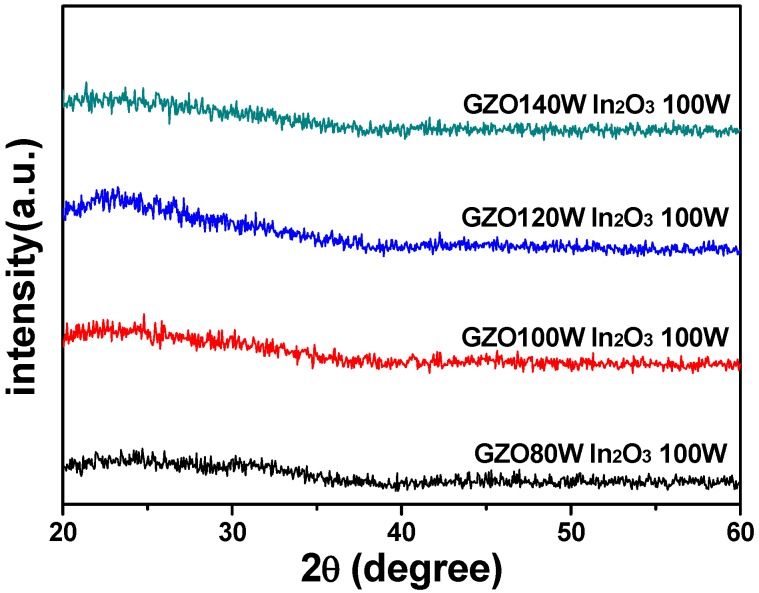
X-ray diffraction (XRD) patterns of In-Ga-Zn-O (IGZO) thin films as a function of deposition power of GZO ceramic target.

As we know, FESEM could be used to observe the surfaces’ crystallization of IGZO thin films. Figure 1 indicates that as deposition power was changed, the surface morphologies of IGZO thin films showed different results. As the deposition power of GZO ceramic target was 80 W or 100 W, the nano-crystalline grains were really observed on the surfaces of IGZO thin films. As the deposition power of GZO ceramic target was increased from 120 W to 140W, surface morphologies of IGZO thin films exhibited a very smooth surface regardless of deposition power of GZO ceramic target. However, most IGZO thin films showed stable and flat amorphous surface features. In order to achieve high performance TCOs-based TFTs or memory devices, the preparation of source and drain electrodes with a smooth surface morphology is very important because surface roughness of IGZO thin films will influence the leakage current between the semiconducting IGZO active layer and source/drain electrodes. The surface observation results suggest that the co-sputtering method is an acceptable method to deposit IGZO thin films, because all IGZO thin films have low roughness surfaces and can be used to fabricate the TCOs-based TFTs or memory devices with high performance.

**Figure 2:**
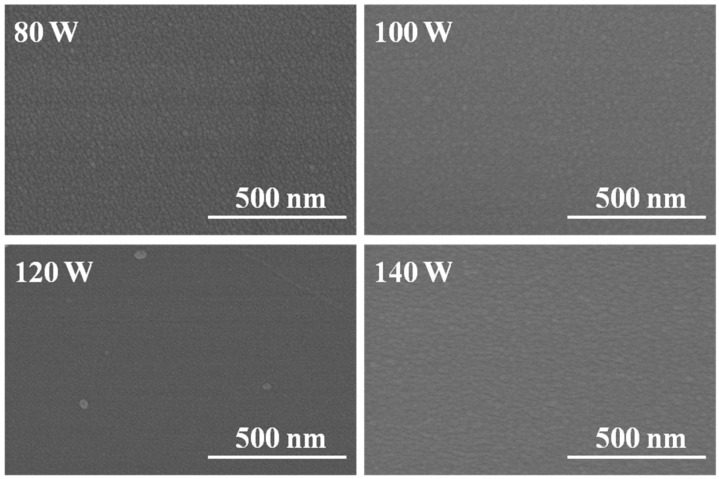
Surface morphology of IGZO thin films as a function of deposition power of GZO ceramic target.

Figure 2 shows the cross-section observations of IGZO thin films as a function of deposition power of GZO ceramic target. As the results in Figure 2 show, thickness of IGZO thin films was around 103 nm, 138 nm, 149 nm, and 170 nm, as the deposition powers of GZO ceramic target was 80 W, 100 W, 120 W, and 140 W, respectively. Thickness of IGZO thin films increasing with deposition power of GZO ceramic target can be fairly expected, because more GZO particles will deposit onto glass substrates to form IGZO thin films. As the cross-session micrographs shown in Figure 2 were compared, there were different results as the deposition power of GZO ceramic target was changed. When the deposition power was 80 W, IGZO thin films grew irregularly. When 100 W and 120 W were used as the deposition powers, IGZO thin films grew like a densified aggregations of nano-laminations and nano-wires with random directions. AWhens the deposition power was 140 W, the aggregations of nano-laminations and nano-wires was changed to nano-wire-aggregated growths, and the nano-wires were highly oriented parallel to the substrate normal. In addition, there is no evidence of the segregation of GZO and In_2_O_3_ due to the uniform co-sputtering of GZO and In_2_O_3_ targets using tilted cathode guns. K. Nomura *et al.* reported that IGZO crystal is composed of alternating stacks of InO_2_^−^ and GaO(ZnO)^+^ layers and the concentration of In_2_O_3_ has a large effect on the crystallization of IGZO thin films [12].

From the standard XRD patterns revealed in JCPD cards, the main crystallization peak of In_2_O_3_ thin films is in the (222) plane [14] and the main crystallization peak of GZO thin films is in the (002) plane [15]. For the InO_2_^−^ layer an In^3+^ ion is located at an octahedral site coordinated by six oxygen and for the GaO(ZnO)^+^ layer Ga^3+^ and Zn^2+^ ions are located at triangle-bipiramidal sites and are each coordinated by five oxygen and alternately stacked along the (0001) direction. Those descriptions suggest that as the deposition power of GZO ceramic target in the co-sputtering method is changed, IGZO thin films have different surface morphology. As the deposition power of GZO target increases, the concentration of In_2_O_3_ decreases, the crystallization direction of GZO will dominate the growth direction of IGZO thin films, then the IGZO thin films have high c-axis orientation and are highly oriented parallel to the substrate normal. As Figure 2 shows, as the deposition power of GZO ceramic target is equal and higher than 120 W, the nano-wires parallel to the substrate normal suggest the stacked along the (002) direction. The structure of nano-laminations and nano-wires is changed to nano-wires parallel to the substrate normal, which proves that GZO thin films will dominate growth results of IGZO thin films. The results observed from the cross-session images of IGZO thin films shown in Figure 2 agree with the results of K. Nomura *et al.* [12] and Wang *et al.* [15].

**Figure 3:**
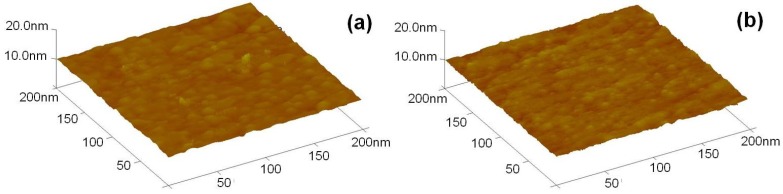
Surface Atomic Force Microscopy (AFM) morphology of IGZO thin films as a function of deposition power of GZO ceramic target. (**a**): 80 W (**b**): 140 W.

AFM images of the two surfaces are presented in Figure 3 and the corresponding roughness values are measured using the described software. It can be noticed from Figure 3 that surface of IGZO thin films for GZO target’s deposition power of 80 W (Figure 3a) is clearly much rougher than that of IGZO thin films for GZO target’s deposition power of 140 W (Figure 3b) as indicated by the Root Mean Square (RMS) roughness values. The RMS roughness values of IGZO thin films’ surface were obtained at five different locations and the average RMS roughness values were determined from the five data. The measured RMS roughness values for Figure 3a were in the range of 2.9 nm~4.5 nm and the average RMS value was 3.8 nm. The measured RMS roughness values for Figure 3b were in the range of 2.2 nm~3.2 nm and the average RMS value was 2.6 nm. 

**Figure 4:**
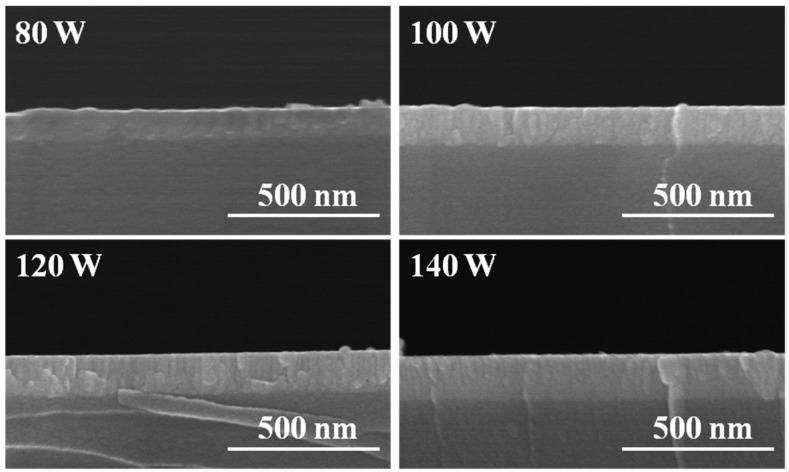
Cross-section observations of IGZO thin films as a function of deposition power of GZO ceramic target.

As the different sintering temperatures are used, differently crystalline phases will be formed in IGZO ceramic targets, and the multi-crystal phases are only observed in IGZO ceramic targets. Lo *et al.*, found cubic Ga_2_ZnO_4_ spinel and rhombohedral InGaZnO_4_ phases are identified in the 1100 °C -sintered sample in addition to the as-prepared oxide powder phases of In_2_O_3_, Ga_2_O_3_, and ZnO [16]. However, most IGZO thin films will reveal the amorphous phase rather than the poly-crystal phases. For example, Jeong *et al.* co-deposited the Ga:In_2_O_3_ and Zn:In_2_O_3_ targets, and they obtained α-IGZO thin films rather than poly-crystal IGZO [11]. Jung *et al.* deposited IGZO thin films by using the facing targets sputtering (FTS) method at room temperature, also the deposited IGZO thin films revealed the amorphous phase [3]. As Figure 4 shows, only one weak and broad peak was assigned to the glass substrate, which proves that all deposited IGZO thin films exhibited the amorphous phase. However, the cubic Ga_2_ZnO_4_ and spinel rhombohedral (poly-crystal) InGaZnO_4_ phases and the phases of precursor In_2_O_3_, Ga_2_O_3_, and ZnO were not observed in the Figure 4.

As we know, energy dispersive spectrometer (EDS) and secondary ion mass spectrometry (SIMS) are generally considered to be the qualitative techniques to find the large variation in ionization probabilities among different materials. For that, we used the two methods to analyze the IGZO thin films and to find the variations of atom ratios at the surface (EDS) and across the depth profile (SIMS) of IGZO thin films. Atomic ratio microanalysis in the FESEM is performed by measuring the energy or wavelength and intensity distribution of X-ray signal generated by a focused electron beam on the specimen. With the attachment of EDS, the precise elemental composition of materials can be obtained with high spatial resolution. Table 1 shows EDS analysis results as a function of deposition power of GZO ceramic target. The atom ratios of Zn and Ga elements increased and atom ratio of In element decreased with increasing deposition power of GZO ceramic target. Those results are expectable because as the deposition power of GZO ceramic target increases, more Ga_2_O_3_ and ZnO (or Ga_2_O_3_ + 2 ZnO) molecules will be moved out from the surface of GZO ceramic target, then atom ratios of Zn and Ga increase and atom ratio of In increases. Table 1 shows important results: even the deposition power of the GZO target is higher than that of In_2_O_3_ target, and the atom ratio of the In element is higher than those of the Ga and Zn elements.

**Table 1:** Atom ratios of Zn, Ga as a function of deposition power of GZO target.

GZO Power	Zn	Ga	In
80 W	3.0	3.6	93.4
100 W	7.0	7.5	85.5
120 W	12.5	12.8	74.7
140 W	19.6	18.3	62.1

**Figure 5:**
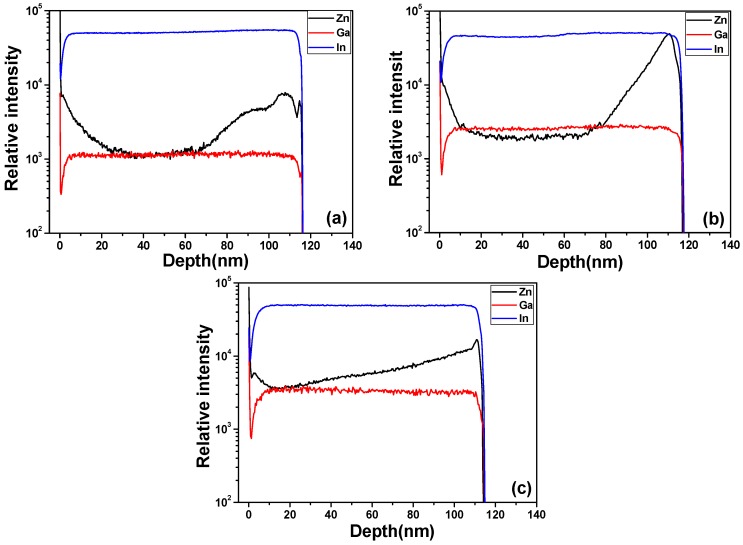
Second ion mass spectrometry analysis of IGZO thin film, the deposition power of GZO ceramic target was (**a**) 80 W (**b**) 100 W and (**c**) 120 W.

Because SIMS is a high sensitivity surface analysis technique for the determination of surface composition and contaminant analysis and for depth profile in the uppermost surface layers of a sample, it can detect very low concentrations of dopants and impurities. For that, the SIMS analysis was used to find the atomics’ concentrations of the constituent elements (In, Ga, and Zn) as a function of the sample’s depth to determine the elemental composition of the surface to a depth of about 120 nm, and the results are shown in Figure 5. IGZO thin films showed that there were incorporations of Zn, Ga, and In atoms in IGZO thin films, even the deposition process was proceeded at room temperature. The concentrations of In and Ga elements in the depth profile was almost unchanged and showed an uniformity distribution, independent of the deposition power of GZO target. However, the results in Figure 5 show that the concentration of Zn element in the depth profile was not uniform distribution. The concentration of Zn element first decreased and then increased as the analyzed depth increased, independent of the deposition power of GZO target.

As the results in Figure 5 are compared, the concentrations of Ga and Zn elements increased and the concentration of In element was almost unchanged as the deposition power of GZO ceramic target increased. The relative In concentration in the depth profile of IGZO thin films shown in Figure 5 is higher than that of the predicted values obtained from the used targets. Those results are very important because so far no SIMS analysis has been used to find the distribution of Zn, Ga, and In elements in the depth profiles of the deposited IGZO thin films. Figure 5 also shows that as the deposition power of GZO ceramic was changed from 80 W to 120 W, the relative concentration of In element was higher than those of Ga and Zn elements, those results are matched the analyzed results show in Table 1.

The reasons to cause the non-uniform distribution of Zn element in the depth profile are not really know. However, during the deposition process, the temperature significantly contributes to precursors decomposition and the growth mechanism and of thin films, and the growth mechanism is strongly depends on the reactor design and process parameters. Saha *et al.*, observed a significant decrease in growth rate and deteriorated structural of the ALD-ZnO films at 250 °C [17]. The higher growth rate might be because of precursor condensation due to their insufficient reactivity to the surface functional groups. Heo *et al.* used Zn as target to grow c-axis oriented ZnO thin films on c-plane Al_2_O_3_ via molecular beam epitaxy (MBE) using dilute ozone (O_3_) as an oxygen source [18]. They found that for growth temperature higher than 350 °C; the rate dramatically decreased and for growth temperatures above 450 °C; continuous films were not realized. They also found that an increase in growth temperature causes a decrease of the sticking coefficient of Zn on the Al_2_O_3_ substrate which, subsequently, causes a decrease in the growth rate, even though the reactivity between Zn and the oxygen source is expected to increase with growth temperature. Those results suggest that using Zn_2_Ga_2_O_5_ ceramic and In_2_O_3_ ceramic to co-deposit IGZO thin films at room temperature, the temperature on the glass substrates is higher, maybe higher than 300 °C; For that, the non-uniform distribution of Zn element in depth profile will be observed.

**Figure 6:**
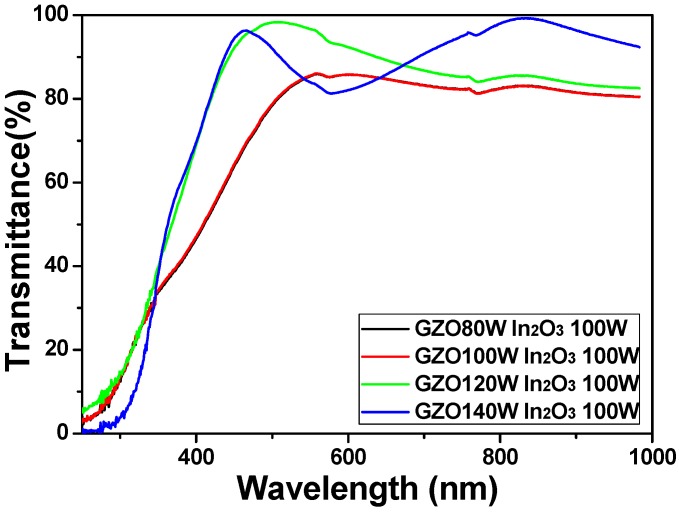
Transmittance spectrum of IGZO thin films as a function of deposition power of GZO ceramic target.

Figure 6 shows the transmittance ratios of IGZO thin films plotted against wavelengths in the region of 250–1000 nm, with deposition power of GZO ceramic target as the parameter. The results in Figure 6 show that the transmittance ratios in the visible light region are apparently changed as the deposition power of GZO ceramic target is changed from 80 W to 140 W. The average transmittance ratio of IGZO thin films in the range of 400 nm~700 nm first increases with deposition power of GZO ceramic target and reaches a maximum value as the deposition power of GZO ceramic target is 120 W. As the deposition power of GZO ceramic target was 80 W, 100 W, 120 W, and 140 W, the average transmittance ratio of IGZO thin films in the range of 400 nm~700 nm was 77.3%, 77.5%, 91.4%, and 86.6%, respectively. Figure 6 also shows that IGZO thin films deposited on glass substrates had the maximum transmittance ratio of over 86.0%, 86.1%, 98.3%, and 96.3% in the range of 400~700 nm as the deposition power of GZO ceramic target was 80 W, 100 W, 120 W, and 140 W, respectively.

Those results suggest that as the co-sputtering method is used, we can deposit IGZO thin films with high transmittance ratio. From the results shown in Figure 1, the surfaces of all deposited IGZO thin films reveal a smooth structure and no agglomerated particles are observed, which are the reasons to cause IGZO thin films having high average transmittance ratio. For the transmission spectra shown in Figure 6, as the different deposition power of GZO ceramic target was used, the shift of the optical band edge was really observable and a greater sharpness was noticeable in the curves of the absorption edge. Those results suggest that the optical band gap (*E_g_*) values will change as the co-sputtering method is used to prepare IGZO thin films.

**Figure 7:**
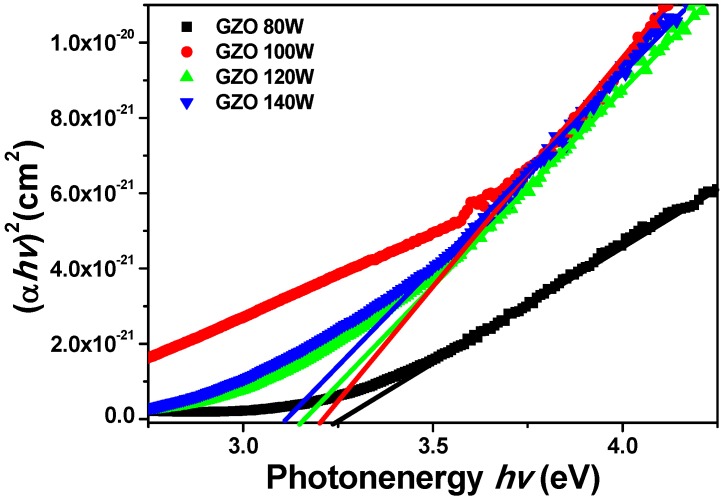
Α^2^ *vs. hν-E_g_* Tauc plots of IGZO thin films as a function of deposition power of GZO ceramic target.

The linear dependence of (α*hv*)^2^ on *hν* indicates that IGZO thin films are direct transition type semiconductors. In accordance with Equation (1), as Figure 7 shows, the calculated *E_g_* values of IGZO thin films were 3.87 eV, 3.84 eV, 3.79 eV, and 3. 71 eV as the deposition power of GZO target were 80 W, 100 W, 120 W, and 140 W, respectively. Because ZnO, Ga_2_O_3_, and In_2_O_3_ thin films have different *E_g_* values, the variation in *E_g_* values is believed to cause by the variation in the composition of IGZO thin films. The *E_g_* values of ZnO [19], In_2_O_3_ [20], and intrinsic β-Ga_2_O_3_ thin films [21] are about 3.40 eV, 3.71 eV, and 4.90 eV, respectively. In general, the measured *E_g_* values of IGZO thin films are consistent with and should be larger than that of ZnO and In_2_O_3_ thin films. However, the *E_g_* values of IGZO thin films do not increase with the increase of deposition power of GZO target, even the atom concentration of Ga element (Ga_2_O_3_) increase, as Figure 7 shows.

In the past, Nomura *et al.*, reported that the In^3+^ can provide extra carriers, when the TFTs devices is fabricated using the In-rich thin films the carrier concentration will be increased. Then, the devices will have larger drain current (I_DS_) and better carrier mobility, and the needed off current will be increased [12]. Kim *et al.*, deposited α-IGZO thin films using the sol-gel method and the In:Ga:Zn mole ratio was controlled as 1:1:2, 3:1:2, and 5:1:2, respectively. They found that the transfer curves are shifted from the positive to the negative direction, *i.e.*, V_th_ of IGZO TFTs decreases from 15.84 V, 4.98 V, to −5.09 V as the In:Ga:Zn mole ratio increases from 1:1:2, 3:1:2, to 5:1:2 [22]. We believe the real In:Ga:Zn mole ratios are not 1:1:2, 3:1:2, and 5:1:2, respectively, but In concentration in IGZO thin films will affect their properties is un-doubtable. Kim *et al.* proved that the electronic concentration and mobility increased and resistivity decreased with increasing In/Ga ratio in IGZO thin films [22]. When IGZO thin films are deposited using the co-sputtering method, three reasons are believed to influence the carrier mobility of IGZO thin films. First, depositing at room temperature cannot provide enough energy to enhance the motion of plasma molecules. Then, the crystallization and grain size growth of IGZO thin films cannot be improved, the defects in IGZO thin films will generate during the deposition process. Second, if the agglomerated particles in IGZO thin films increase, that will cause the increase in the inhibiting of the barriers electron transportation and the mobility will decrease. Third, H. Hosono showed that the electron mobility and concentration evaluated from the Hall effects for α-IGZO thin films with different compositions, the mobility is primary determined by the fraction of In_2_O_3_ concentration and the highest value of ~40 cm^2^ (V·s)^−1^ is obtained around the samples containing the maximum In_2_O_3_ fraction [23]. From those reasons, the carrier mobility, carrier concentration, and resistivity of IGZO thin films are believed to be dependent on deposition power (or concentration) of GZO target.

**Figure 8:**
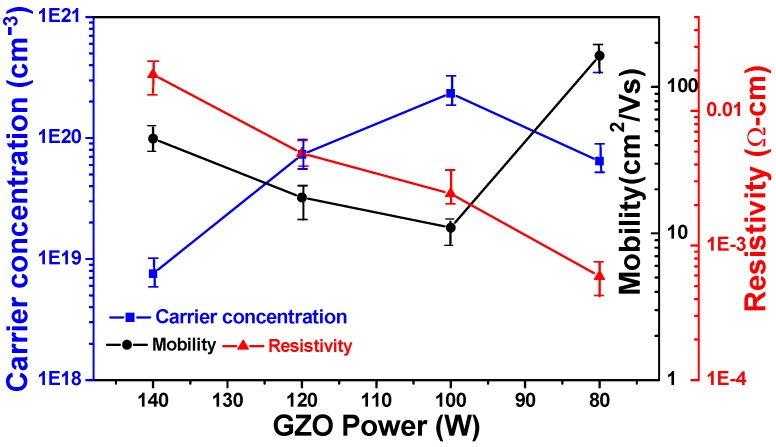
Hall mobility, carrier concentration, and resistivity of IGZO thin films as a function of deposition power of GZO ceramic target.

In this study, at least five Hall-effect coefficients of IGZO thin films were measured for each deposition parameter, and the average values with the deviation ranges were shown in Figure 8. However, we obtained the different results as compared with those of Kim *et al.*, [22] and H. Hosono [23]. As Figure 8 indicates that as the deposition power of GZO ceramic target was 80 W, 100 W, 120 W, and 140 W, the carrier concentration was 6.45 × 10^19^ cm^−3^, 2.34 × 10^2^^0^ cm^−3^, 7.30 × 10^19^ cm^−3^, and 7.57 × 10^18^ cm^−3^, and the carrier mobility was 163.4 cm^2^/V-s, 11.0 cm^2^/V-s, 17.6 cm^2^/V-s, and 44.4 cm^2^/V-s, respectively. There are two reasons are believed to cause IGZO thin films having a high mobility of 163.4 cm^2^/V-s. The first is the high In ratio in the IGZO thin films formed for GZO ceramic target with a deposition power of 140 W. From the EDS and SIMS analyses results in Table 1, the In ratio decreased with the increase of deposition power of GZO target. Those results suggest that the concentration of In_2_O_3_ is the most important factor to influence the mobility of IGZO thin films and the results agree with the important results investigated by H. Hosono [23]. Generally, the field-effect mobility of semiconductor thin films of TFT devices is determined by many factors, including the energy band properties of the active layers and the interface states [24]. The related energy band states of the active layers involve deep states, band-tail states, and extended states. The second reason suggests that Hall mobility of IGZO thin films scarcely increases with the increase in deposition power of GZO ceramic target because the deep states and tail-like states in α-IGZO show little dependence on RF power. The resistivity of TCO thin films is proportional to the reciprocal of the product of carrier concentration N and mobility μ:

ρ = 1/Neμ
(2)

Both the carrier concentration and the carrier mobility contribute to the conductivity. As the deposition power of GZO ceramic target was changed from 80 W to 140 W, the resistivity of IGZO thin films was linearly increased from 5.91 × 10^−4^ Ω-cm to 1.86 × 10^−2^ Ω-cm. The minimum resistivity of IGZO thin films at a deposition power of GZO ceramic target of 80 W is mainly caused by the carrier mobility at its maximum.

## 4. Conclusions

The characteristics of IGZO thin films prepared by Ga_2_O_3_-2 ZnO (GZO) and In_2_O_3_ co-sputtering method were well investigated in this study. As the deposition powers of GZO ceramic target was 80 W, 100 W, 120 W, and 140 W, the thickness of IGZO thin films was around 103 nm, 138 nm, 149 nm, and 170 nm; the average transmittance ratio of IGZO thin films in the range of 400 nm~700 nm was 77.3%, 77.5%, 91.4%, and 86.6%; and the calculated *E_g_* values of IGZO thin films were 3.87 eV, 3.84 eV, 3.79 eV, and 3.71 eV, respectively. From the SIMS analysis results of IGZO thin films, the concentrations of In and Ga elements in the depth profile showed an uniformity distribution and the concentration of Zn element in the depth profile was not uniform distribution. As the deposition power of GZO thin films increased, the concentrations of Ga and Zn elements increased in the depth profile and the concentration of In element in the depth profile was almost unchanged. As the deposition power of GZO ceramic target increased from 80 W to 140 W, the carrier mobility of IGZO thin films was in the range of 11.0 cm^2^/V-s~163.4 cm^2^/V-s and the resistivity of IGZO thin films was linearly increased from 5.91 × 10^−^^4^ Ω-cm to 1.86 × 10^−^^2^ Ω-cm. The mobility of 163.4 cm^2^/V-s is higher than those of most reported IGZO thin films.
